# Value of 3D ultrasound flow imaging combined with serum AFP, β-hCG, sFlt-1 and CK in the diagnosis of placenta accreta

**DOI:** 10.1186/s12905-022-02107-z

**Published:** 2022-12-29

**Authors:** Sheng-nan Cai, Yan-ting Wu, Li Zeng, Yi-qian Ding

**Affiliations:** Department of Gynecology, Affiliated Matern&Child Care Hospital of Nantong University, 399 Century Avenue, Chongchuan District, Nantong, 226000 Jiangsu China

**Keywords:** Placenta accrete, AFP, β-hCG, sFlt-1, CK, Three-dimensional ultrasound, Combined prediction, Diagnostic value

## Abstract

**Purpose:**

To analyze the diagnostic value of placenta three-dimensional (3D) energy blood flow parameters combined with maternal serum AFP, β-hCG, sFlt-1 and CK levels for PA.

**Methods:**

30 pregnant women with PA and 30 pregnant women with normal placenta were randomly selected in the Affiliated Maternal and Child Health Hospital of Nantong University from January 2021 to December 2021. Thereafter, the 3D energy ultrasound was applied to detect the placenta VI, FI and VFI. Moreover, the diagnostic value of different parameters combined with serum AFP, β-hCG, sFlt-1 and CK levels for PA was analyzed.

**Results:**

Multivariate analysis results indicated that, gravidity > 2 and with/without placenta previa were the independent risk factors for PA (*P* < 0.05). In PA group, the AFP, β-hCG, CK, placenta VI, FI and VFI values were higher than those in non-PA group, while sFlt-1 was apparently lower than that in non-PA group. With the increase in PA degree, the serum AFP, β-hCG and CK levels increased. Meanwhile, serum sFlt-1 level was negatively correlated with PA degree. Serum AFP, β-hCG, sFlt-1, CK and placenta VFI showed prediction potency for PA, and their combined detection attained the optimal diagnostic value for predicting PA. ROC curve analysis suggested that, serum AFP, β-hCG, sFlt-1, CK and 3D ultrasound VFI value had the greatest AUC values in predicting PA, which might provide reference for the clinical diagnosis and disease evaluation of PA. Conclusion Serum AFP, β-hCG, sFlt-1, CK and placental VFI can increase the consistency in the diagnosis of PA. Serum markers combined with 3D ultrasound blood flow imaging can improve the sensitivity and specificity of prenatal diagnosis of PA, which provides an important reference for clinical diagnosis and treatment.

## Introduction

Placenta accreta (PA) is an obstetric emergency, which is likely to induce severe complications like uterine rupture, massive hemorrhage, and secondary infection, increases the perinatal hysterectomy rate, and is the major cause leading to maternal death [1]. According to the invasion depth of placental villus into myometrium, PA is classified as placenta accreta, placenta increta and placenta percreta [2]. In recent years, the increased cesarean section rate, the opening of the two-child policy, the increased elderly maternal number, the rapidly increased intrauterine operations like induced abortion and hysteroscopy, the use of contraceptives, the increased pelvic infection rate, and myomectomy are the high risk factors, which have resulted in endometrial injury to varying degrees and provided conditions for the occurrence of PA. Prenatal ultrasound is the preferred option to diagnose PA. In particular, three-dimensional (3D) ultrasound displays numerous advantages such as high-resolution plane image analysis, 3D imaging, easy operation and non-invasiveness, and it has improved the prenatal detection rate of PA. However, artifact may occur in 3D ultrasound, which interferes with the correct image display and results in misdiagnosis [3]. Therefore, more accurate detection means are needed to improve the prenatal diagnosis rate of PA. Some research suggests that the occurrence and development of PA are accompanying with the abnormalities of multiple cytokine levels, which can compensate for the drawback of 3D ultrasound flow imaging when it is applied in prenatal PA screening. On the other hand, maternal serological examination exhibits the advantages like less trauma and reproducibility, which is an important auxiliary method commonly used to diagnose disease in clinic. Typically, serum alpha fetoprotein (AFP), free-β human chorionic gonadotropin (β-hCG), soluble Fms-like tyrosinekinase-1 (sFlt-1) and creatine kinase (CK) are the important indexes for prenatal screening [4–6]. This study combined 3D energy ultrasonic parameters with maternal serum AFP, β-hCG, sFlt-1 and CK levels in the diagnosis of PA, so as to provide reference for improving the prenatal detection rate of PA in clinic, and lay certain foundation for developing the new method and pathway of clinical diagnosis and treatment.

## Materials and methods

### Research object

From January 2021 to December 2021, clinical data from 60 pregnant women giving birth at the Affiliated Maternity and Childcare Hospital of Nantong University were selected. All cases were divided into PA groups (n = 30) and non-PA groups (n = 30) according to intraoperative or intrapartum diagnosis. The case inclusion criteria were as follows, ① for PA group, cases with postnatal incomplete placenta or placental retention that could hardly be stripped; ② cases diagnosed based on MRI and pathology; ③ the criteria of PA [7]: placenta accreta, placenta increta and placenta percreta types; ④ singleton pregnancy; and ⑤ pregnant women with stable vital signs. The case exclusion criteria were shown below, ① those with combined pregnancy complications like gestational diabetes mellitus (GDM) and gestational hypertension; ② those with autoimmune disease, hematological disease, and severe liver, kidney and heart dysfunction; and ③gemellary or multiple pregnancies. This study was approved by the hospital ethics committee. All research objects signed the informed consent.

### Research methods and indexes

The general informations for the samples were obtained from the maternal file information when the pregnant woman first came to the hospital.

Sample collection. Before delivery, 4 mL fasting venous blood was collected from each research object and centrifuged to collect the serum. Thereafter, the serum samples were stored at − 4 °C.

Measurements of serum AFP, β-hCG, sFlt-1 and CK levels. The serum AFP content was measured by electrochemiluminescence (ECLIA) method, where the β-hCG, sFlt-1 and CK levels were detected using the enzyme-linked immunosorbent assay (ELISA) kits, respectively.

3D ultrasound flow imaging examination: The placental location, thickness and invasion depth in the myometrium were observed with two-dimensional (2D) ultrasound. Thereafter, the 3D volume transducer was utilized to observe the posterior placental space and invasion depth of the placenta in myometrium, and to collect the blood vessel images. Attention should be paid to maintain the fixed position of the transducer during the image collection process (time, 10–15 s). Then, the data were saved. Afterwards, the VOCAL software was employed to measure the 3D power doppler ultrasonography (3D-PDU) indexes, including vascularization index (VI), flow index (FI), and vascularized flow index (VFI).

### Statistical analysis

The statistical methods were shown below. The SPSS26.0 software was utilized for statistical analysis of the collected data. Measurement data were described as ${\overline{\text{x}}}$ ± S. Independent sample t-test was adopted for comparison between two groups, and chi-square test was applied in comparison among multiple groups. Risk factors were analyzed with the logistic regression analysis model. The receiver operating characteristic (ROC) curve was plotted to analyze the prediction performance, meanwhile, the area under the curve (AUC) value, confidence interval (CI), sensitivity, specificity and cut-off value were also obtained. DeLong test was applied to compare AUC values among different prediction schemes. A difference of *P* < 0.05 stood for statistical significance.

## Results

### Comparison of general data

Differences in the pre-pregnancy body mass index (BMI) and intrapartum gestational week between PA groups and non-PA groups were not significant (*P* > 0.05). Meanwhile, other general data, including age, gravidity, induced abortion times, history of caesarean section, history of intrauterine surgery, and with/without placenta previa were significantly different between two groups (*P* < 0.05). More details can be obtained from Table 1.

**Table 1:** Comparisons between general data between PA groups and non-PA group

Group factor	PA group (n = 30)	Accreta (n = 10)	Increta (n = 14)	Percreta (n = 6)	Non-PA group (n = 30)	χ^2^	*P*
*Age*						4.356	0.037
Age < 35 years			19 (63.33)		26 (86.67)		
Age ≥ 35 years			11 (36.67)		4 (13.33)		
*Pre-pregnancy BMI*						0.884	0.347
≤ 24			22 (73.33)		25 (83.33)		
> 24			8 (26.67)		5 (16.67)		
*Gravidity*						4.444	0.035
≤ 2			8 (26.67)		16 (53.33)		
> 2			22 (73.33)		14 (46.67)		
*Induced abortion times*						16.710	0.000
≤ 1			15 (50.00)		29 (96.67)		
> 1			15 (50.00)		1 (3.33)		
*History of cesarean section*						4.593	0.032
Yes			15 (50.00)		7 (23.33)		
No			15 (50.00)		23 (76.67)		
*History of intrauterine surgery*						4.320	0.038
Yes			8 (20.00)		2 (6.67)		
No			22 (80.00)		28 (93.33)		
*Placental previa*						10.335	0.001
Yes			17 (56.67)		5 (16.67)		
No			13 (43.33)		25 (83.33)		
*Intrapartum gestational week*						1.832	0.176
< 37 weeks			12 (60.00)		8 (26.67)		
≥ 37 weeks			18 (40.00)		22 (73.33)		

Affiliated Matern&Child Care Hospital of Nantong University, 2021

### Influencing factors for PA occurrence

PA occurrence was treated as the dependent variable, whereas age, gravidity, history of cesarean section, history of intrauterine surgery, and with/without placenta previa as the independent variables. Upon logistic regress analysis, gravidity > 2 and placenta previa were the independent risk factors for PA occurrence (*P* < 0.05, Table 2).

**Table 2:** Multivariate logistic regression analysis of PA

Factor	β	SE	Wald χ^2^	OR	95% CI	*P*
Age	0.066	0.080	0.680	1.068	0.913–1.249	0.410
Gravidity	1.073	0.399	7.234	2.923	1.338–6.389	0.007
History of cesarean section	-0.395	0.798	0.245	0.674	0.141–3.217	0.620
History of intrauterine surgery	1.399	1.076	1.689	4.049	0.491–33.366	0.194
Placenta previa	1.634	0.777	4.421	5.126	1.117–23.518	0.036

Affiliated Matern&Child Care Hospital of Nantong University, 2021

### Comparisons of serum AFP, β-hCG, sFlt-1 and CK levels among placenta accreta, placenta increta, placenta percreta and non-PA groups

The serum AFP, β-hCG and CK levels followed the order of placenta percreta group > placenta increta group > placenta accreta group > non-PA group. With regard to serum sFlt-1 level, it followed the order of placenta percreta group < placenta increta group < placenta accreta group < non-PA group. Differences were of statistical significance (*P* < 0.05, Table 3).

**Table 3:** Comparisons of serum indexes among the four groups (${\overline{\text{x}}}$ ± s)

Serum index grouping	AFP (U/mL)	β-hCG (ng/mL)	sFl-t (μg/L)	CK (U/L)
Placenta accreta (n = 10)	127.33 ± 10.37	266.54 ± 11.95	2735.71 ± 656.55	116.30 ± 7.38
Placenta increta (n = 14)	128.45 ± 0.39	316.95 ± 10.67	2348.51 ± 69.92	119.81 ± 34.50
Placenta percreta (n = 6)	140.12 ± 9.89	439.61 ± 41.72	2075.57 ± 166.13	149.21 ± 22.82
Non-PA groups (n = 30)	111.62 ± 14.25	224.67 ± 59.79	2866.41 ± 226.77	85.78 ± 1.40
χ^2^	13.01	16.69	15.99	25.86
*P*	0.005	0.001	0.001	0.000

Affiliated Matern&Child Care Hospital of Nantong University, 2021

### Ultrasound flow parameters of PA groups and non-PA group

The VI, FI and VFI levels in PA groups were hither than those in non-PA group, the differences in VI and FI were not statistically significant (*P* > 0.05), while the difference in VFI was of statistical significance (*P* < 0.05, Table 4).

**Table 4:** Comparisons of ultrasound flow indexes between PA groups and non-PA groups (${\overline{\text{x}}}$ ± s)

Ultrasound index grouping	VI	FI	VFI
PA groups (n = 30)	27.92 ± 10.78	38.87 ± 1.94	13.45 ± 3.34
Non-PA groups (n = 30)	24.59 ± 0.33	33.30 ± 3.95	9.08 ± 0.39
t	5.46	9.145	6.454
*p*	0.105	0.08	0.01

Affiliated Matern&Child Care Hospital of Nantong University, 2021

### Prediction value of serum indexes and ultrasound flow parameters for PA

**Fig. 1:**
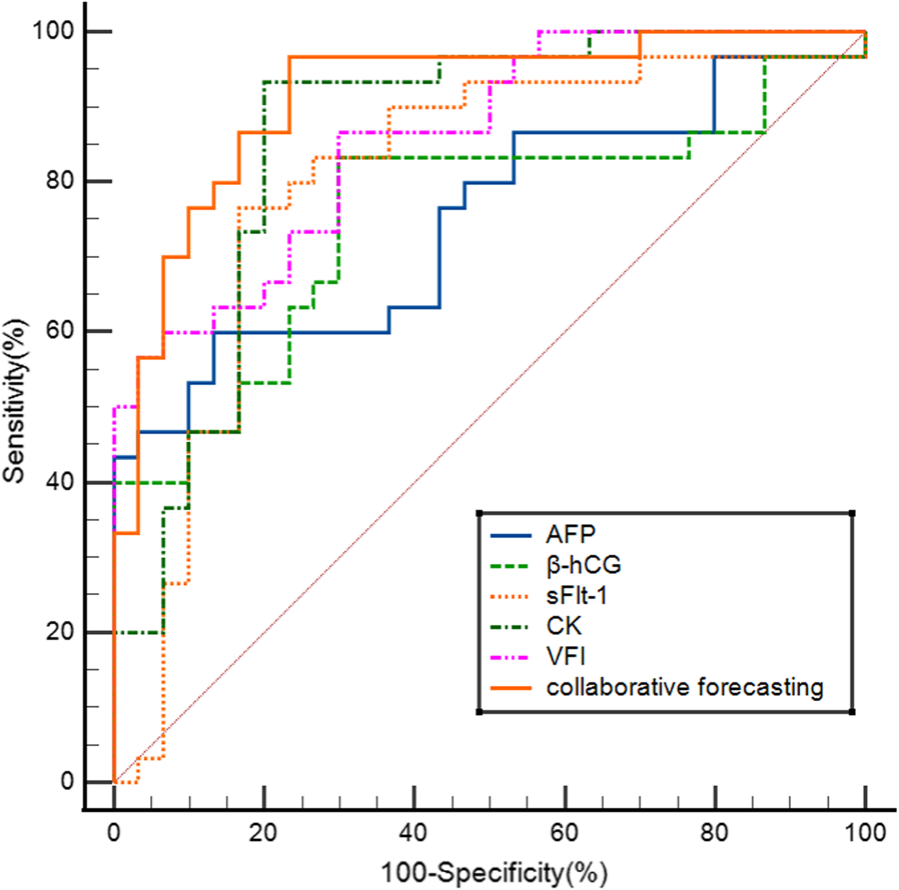
ROC curves of PA prediction plotted based on serum indexes and 3D ultrasound flow parameters

The ROC curves of PA prediction were plotted based on the serum AFP, β-hCG, sFlt-1 and CK levels and 3D ultrasound flow parameter VFI value (Fig. 1). As a result, CK had the highest AUC value, followed by VFI, sFlt-1, β-hCG and AFP. The AUC value of serum indexes combined with 3D ultrasound flow parameters in predicting PA was 0.916, while the 95% CI, sensitivity, specificity, positive prediction rate, negative prediction rate and accuracy were 0.815–0.973, 96.67%, 76.67%, 90.00%, 80.00% and 85.00%, respectively (Table 5).

**Table 5:** Prediction value of serum AFP, β-hCG, sFlt-1 and CK combined with 3D ultrasound VFI for PA

	AUC	P	Youden index	Cut-off value	Sensitivity	Specificity	Positive prediction rate	Negative predicti on rate	Accuracy	95% CI
AFP	0.749	< 0.001	0.467	127.55	60.00	86.67	60.00	83.33	71.67	0.620–0.852
β -hCG	0.754	< 0.001	0.533	221.45	83.33	70.00	83.33	66.67	75.00	0.626–0.856
sFl-t	0.800	< 0.001	0.600	2630.34	76.67	83.33	76.67	90	83.33	0.677–0.892
CK	0.866	< 0.001	0.733	86.67	93.33	80.00	90.00	76.67	83.33	0.745–0.935
VFI	0.870	< 0.001	0.600	10.34	83.33	76.67	83.33	73.33	78.33	0.758–0.943
Collaborative forecasting	0.916	< 0.001	0.700		96.67	76.67	90.00	80.00	85.00	0.815–0.973

Affiliated Matern&Child Care Hospital of Nantong University, 2021

## Discussion

The morbidity of PA shows an increasing trend year by year, which has become one of the major causes leading to perinatal hysterectomy, postpartum hemorrhage and maternal death. However, the pathogenic mechanism of PA remains unclear, which may be related to decidua dysplasia, enhanced invasiveness of trophoblasts and changed placental angiogenesis [7]. The high risk factors for PA include advanced age, history of cesarean section, history of intrauterine operation, history of multiple pregnancies and births and placenta previa. In this study, the general data of patients with PA were statistically analyzed. As a result, age, gravidity, induced abortion times, history of cesarean section, history of intrauterine operation and with/without placenta previa were the important factors for PA occurrence, among which, gravidity and placenta previa were the independent risk factors.

Accurate prenatal evaluation and prediction of PA type and severity can reduce the occurrence of adverse pregnancy outcome of PA, so as to make adequate preoperative preparation. Multidisciplinary (obstetricians with PA management experience, anesthesiology, neonatology and ultrasonography) cooperation and sufficient preparation of blood source can reduce the potential maternal and neonatal morbidity and mortality rates [8]. 3D ultrasound flow imaging can provide quantitative reference based on the 3D structure and blood flow perfusion at the lesion site, which is commonly used in the clinical diagnosis of PA [9]. However, there is no uniform standard for diagnosis at present, which makes it impossible to effectively predict the PA severity. In addition, the presence of artifact may lead to a certain rate of misdiagnosis. Serological indexes combined with 3D ultrasound flow imaging can provide a new direction for the clinical diagnosis of PA severity.

Alpha fetoprotein (AFP) and β-human chorionic gonadotropin (β-hCG): AFP is the most commonly seen globulin in fetal serum, which is used to evaluate the placenta barrier function. β-hCG is produced by the trophoblasts, which mainly enter the maternal blood, but its content is relatively low in placental tissue. Its detection rate is high and stable in the second trimester of pregnancy, which can reflect the activity of trophoblasts [2]. Some research discovers that the risk of PA increases significantly when the AFP or free β-hCG (fβ-hCG) level is ≥ 2.5 MOM in the second trimester of pregnancy, and the increase amplitude is related to the severity of PA [10, 11]. This study discovered that the serum AFP and β-hCG levels in PA patients were higher than those of non-PA group. Besides, inter-group analysis among PA groups suggested that, the serum AFP and β-hCG levels in patients increased with the increase in PA severity. These results indicate that serum AFP and β-hCG levels are valuable in the diagnosis of placenta accreta. It is possible that, due to decidua developmental defect in PA patients, the trophoblasts invade the myometrium, at this time, AFP in fetal blood enters the maternal circulation, leading to the increased serum AFP levels in pregnant women. The reason for the abnormal increase of β-hCG may be that when PA occurs, placental villi cannot form a good exchange of maternal and infant nutrients with the uterine basement membrane, resulting in hypoxia of placental tissue, resulting in excessive secretion of β-hCG, and finally the increase of its level.

Soluble Fms-like tyrosinekinase (sFlt) is the soluble form of VEGFR-1, which is selectively expressed in placental tissues and is a kind of placenta-specific protein [5]. sFlt-1 can irreversibly bind to VEGF, thus suppressing the physiological function of VEGF. In addition, it exerts the anti-angiogenic activity, which induces endothelial cell dysfunction, and breaks the vascular wall permeability and integrity [12]. It is also speculated that sFlt-1 may play an important role in enhancing the excessive invasion of trophoblasts and vascular remodeling, and this series of changes may be secondary to the endometrial-myometrial injury microenvironment of PA patients [7]. It was found in this work that, the peripheral blood sFlt-1 levels in PA patients were apparently reduced compared with non-PA group, in particular for placenta percreta. Such result was consistent with the PA serological index research results obtained by Schwickert et al. [13]. The analysis of the reason is that VEGF and sFlt-1 in normal pregnant women in the third trimester are in a relatively stable state. When the expression of VEGF increases and sFlt-1 decreases, it promotes the formation and development of placental blood vessels in pregnant women, and then promotes the occurrence and development of PA.

Creatine kinase (CK) mainly exists in the muscle and brain tissue of human body, which is a kinase related to intercellular energy transfer and muscle contraction. When PA occurs, the villi of the placenta invade the Uterine basal layer and damage the muscle cells, CK in the cells is released into the blood, which can elevate the serum CK level After the damage of muscle cells [14]. In this study, the serum CK level was positively correlated with the PA degree, which might serve as the serological index for the prenatal diagnosis of PA. However, the diagnostic value of serum CK in PA remains controversial [6, 15].

3D ultrasound flow imaging can evaluate placenta angiogenesis from the perspectives of VI, FI and VFI, and assess placenta status from the level of blood supply. The VI, FI and VFI values increase in the case of PA, and the increase amplitudes are positively related to the PA degree. But ultrasound imaging can hardly display the placenta when it is located in the posterior wall, and is subject to the experience of the diagnostic physician. According to our research results, the VI, FI and VFI values in PA groups were higher than those in non-PA group. This was mainly because that, a large amount of new blood vessels were formed in the case of PA, which manifested as the elevated VI, FI and VFI values, but only the difference in VFI value was of statistical significance, and it might be related to our low sample size. ROC curve analysis suggested that, serum AFP, β-hCG, sFlt-1, CK and 3D ultrasound VFI value had the greatest AUC values in predicting PA, which might provide reference for the clinical diagnosis and disease evaluation of PA.

Due to the small sample size of the data analyzed in this study, the results of the study may lack corresponding convincing. In the future, studies with a larger sample size are needed to further widely apply the studied indicators to clinical diagnosis.

## Conclusion

To sum up, for pregnant women with PA, the serum AFP, β-hCG, sFlt-1 and CK levels are abnormal. For pregnant women with high-risk factors for PA in the third trimester of pregnancy, the combined application of serum AFP, β-hCG, sFlt-1 and CK with 3D ultrasound flow imaging examination can improve the diagnostic efficacy of prenatal diagnosis of PA, contribute to the judgement of PA type and provide important reference for clinical diagnosis and treatment. It is recommended that the combined detection of serum and ultrasound indicators can provide more comprehensive and reliable reference information for the clinical prediction of PA rate.

## Data Availability

All data generated or analysed during this study are included in this published article.

## Abbreviations

PA: Placenta accreta
3D: Three-dimensional
AFP: Alpha fetoprotein
β-hCG: β-Human chorionic gonadotropin
sFlt-1: Soluble Fms-like tyrosinekinase
CK: Creatine kinase
VI: Vascularization index
FI: Flow index
VFI: Vascularized flow index
